# CAR T-cell therapy for neurological disorders: scientific rationale and mechanistic insights

**DOI:** 10.1177/17562864251396039

**Published:** 2025-12-10

**Authors:** Dimitrios E. Katsaros, Dimitrios Mougiakakos

**Affiliations:** Department of Hematology, Oncology, and Cell Therapy, Otto-von-Guericke University Magdeburg, Magdeburg, Germany; Department of Hematology, Oncology, and Cell Therapy, Otto-von-Guericke University Magdeburg, Leipziger Str. 44, Magdeburg 39120, Germany

**Keywords:** autoimmune diseases, B cell depletion, CAR, cell therapy, neuroimmunology, reset

## Abstract

The development and successful clinical application of engineered T cells expressing synthetic chimeric antigen receptors (CARs) represents a milestone in cancer therapy. This approach optimizes physiological in vivo T-cell activation against specific target antigens expressed on defined cell subsets with the goal of their deep and sustained depletion. Significant progress has been made in redesigning CAR T-cell constructs to improve patient safety, therapeutic efficacy, and accessibility. Efforts have also focused on streamlining manufacturing to improve availability and reduce costs, two critical challenges to widespread adoption. Beyond hematologic malignancies, CAR T-cell therapies are now increasingly being repurposed to tackle B-cell-mediated autoimmune diseases (AIDs). This is primarily achieved through broad B-cell depletion, but more targeted approaches—such as the selective elimination of autoantibody-producing B-cell subpopulations—are also being explored. Important considerations in their implementation are identifying the most pertinent patient groups, tailoring their treatment up to the point of CAR-infusion, and following up on their unique toxicity-profile. In the context of neurological AIDs—including refractory myasthenia gravis, Lambert–Eaton syndrome, multiple sclerosis, and stiff-person syndrome—early clinical experience suggests promising efficacy and tolerability, leading to a growing number of registered clinical trials. In this review, we provide an overview of the mechanism and evolution of CAR T-cell therapy, highlighting why its application in AIDs, particularly in neurology, represents a highly promising therapeutic strategy.

## Plain language summary

How CAR T-cell therapy could help treat neurological disorders

CAR T-cell therapy has revolutionized cancer treatment by engineering a patient’s own immune cells to recognize and destroy harmful cells. Initially developed for blood cancers, this therapy is now being explored for autoimmune diseases, particularly neurological disorders like multiple sclerosis, myasthenia gravis, and stiff-person syndrome. The main way CAR T-cells work in autoimmune diseases is by targeting and eliminating B cells, which play a key role in driving these conditions. Unlike traditional treatments, which often require continuous medication, CAR T-cell therapy has the potential to reset the immune system with long-lasting effects. Early clinical experiences have been promising, showing that CAR T-cell therapy can lead to deep and sustained disease remission. Importantly, the therapy appears to be well tolerated, with fewer severe side effects than in cancer patients. Research is now focusing on optimizing safety, accessibility, and effectiveness to bring this innovative therapy to more patients. By learning from cancer immunotherapy, scientists aim to refine CAR T-cell treatment for neurological autoimmune diseases, potentially offering a transformative new option for patients who have not responded to conventional therapies.

## Introduction

The introduction of biosynthetically engineered chimeric antigen receptor (CAR) T-cells in the treatment of hemato-oncological disorders has had a transformative impact.^1^ Difficult to treat patients with relapsed or refractory (r/r) aggressive B-cell lymphoma achieved deep and long-lasting remissions highlighting the curative potential of this therapy.^2^ One of the very first clinical trials in r/r diffuse large B-cell lymphoma (i.e., ZUMA-1)^3^ increased the patients’ 2-year overall survival to 50% as compared to 17% in historical controls.^4^ Consequently, every year new commercial CAR T-cell products gain regulatory approval for new indications and CAR T-cells already in use are approved for being used at earlier lines. At the same time, significant progress is being made both in the design of next-generation CAR constructs and in the use of alternative cell types, such as NK-cells or macrophages, instead of T-cells. The next major breakthrough is expected with the introduction of allogeneic cell products, which aim to improve accessibility to the still highly expensive CAR T-cell therapies. Moreover, the results in solid tumors remain insufficient, and extensive research and development efforts are focused on overcoming the barriers imposed by the tumor microenvironment (TME). Approaches include genetically modifying CAR cells to secrete cytokines such as interleukin-12 (IL-12) or using mRNA-based vaccines for repetitive restimulation of CAR T cells.^5,6^ Overall, this presents a promising outlook for bench-to-bedside pharmaceutical optimization and signals a productive dialog among multidisciplinary teams. Due to their unique ability to achieve deep depletion of target cells even within inflammatory niches, CAR T cells are currently being repurposed for use in autoimmune diseases (AIDs). What started with a few case reports has rapidly evolved, and by 2025, more than a hundred active clinical trials will be underway—further highlighting the potentially transformative impact of this approach. Although AIDs are not always acutely life-threatening, they significantly impair quality of life, lead to irreversible organ damage, necessitate lifelong use of often potent immunosuppressive medications, and impose a substantial socioeconomic burden.^7^ In this review, we will explore the evolution of cellular therapies leading to CAR T-cells and explain why CAR T-cell therapy might hold great promise for AIDs, including those within the neurological spectrum.

## Literature search

A literature search was performed in the PubMed database. There were no language or publication date restrictions. Only studies in humans that used CAR therapy in any of its forms in patients with autoimmune neurological disorders (including multiple sclerosis (MS), neuromyelitis optica spectrum disorder, myelin oligodendrocyte glycoprotein antibody-associated disease, myasthenia gravis (MG), Lambert–Eaton myasthenic syndrome, stiff-person syndrome, autoimmune peripheral neuropathies, and autoimmune encephalitis) were included. The last search date was August 11, 2025. In total, 2686 publications were screened, which provided individual data on cumulatively 63 patients with a neurological AID within the scope of this review. The identified published studies with the patient data are depicted in Table 1.^8–24^

**Table 1.** Published studies describing CAR T-cell application in autoimmune neurological diseases.

Publication	Study type	Disease	Antigen target	Patients (*n*)	CRS (*n*, grade)	ICANS (*n*, grade)	Construct
Qin et al.^8^	Phase I	NMOSD	BCMA	12	12, I–II	0	CT103A^a^
Granit et al.^9^	Phase Ib/IIa	MG	BCMA	14	0	0	Descartes-08^b^
Haghikia et al.^10^	Case report	MG	CD-19	1	0	0	KYV-101^c^
Tian et al.^11^	Case report	MG	BCMA	2	1, I	0	CT103A^a^
Zhang et al.^12^	Case report	CIDP	BCMA CD-19	1	1, I	0	Bispecific, human, 4-1BB
Fischbach et al.^13^	Case report	MS	CD-19	2	1, I	0	KYV-101
Motte et al.^14^	Case report	MG with LEMS	CD-19	2	2, I–II	1, I	KYV-101
Faissner et al.^15^	Case report	SPS	CD-19	1	1, II	0	KYV-101
Zhang et al.^16^	Case report	MG	BCMA CD-19	1	0	0	Bispecific, human, 4-1BB
Cabrera-Maqueda et al.^17^	Case report	MOGAD	CD-19	1	0	0	ARI-0001^d^
Wickel et al.^18^	Case report	LEMS	CD-19	1	1, II	0	KYV-101
Haghikia et al.^19^	Case report	MG with RA	CD-19	1	1, I	0	KYV-101
Vu et al.^20^	Phase IIb	MG	BCMA	18	0	0	Descartes-08
Dong et al.^21^	Case report	CIDP	BCMA	2	2, I	0	CT103A^a^
Pecher et al.^22^	Case report	MS with RA	CD-19	1	1, II	0	Murine, 4-1BB
Motte et al.^23^	Case report	APN	CD-19	2	2, I–II	1, I	KYV-101
Hegelmaier et al.^24^	Case report	AIE	CD-19	1	1, I	N/A	KYV-101
Total	–	–	–	63	26, I–II	2, I	–

All mentioned CAR products utilize an scFv extracellular domain, a CD8a hinge region, and a CD3ζ signaling domain.

a CT103A consists of a human scFv and 4-1BB as a co-stimulatory domain.

b Descartes-08 consists of a murine scFv, CD28 as co-stimulatory molecule, and is an RNA CAR T-cell construct, administered on a weekly regimen for a set period of time.

c KYV-101 consists of a human scFv and CD28 as co-stimulatory molecule.

d ARI-0001 consists of a A3B1-derived murine scFv and 4-1BB as co-stimulatory molecule.

AIE, autoimmune encephalitis; APN, autoimmune peripheral neuropathy; BCMA, B-cell maturation antigen; CAR, chimeric antigen receptor; CIDP, chronic inflammatory demyelinating polyneuropathy; CR, case report; CRS, cytokine release syndromes; ICANS, immune effector cell-associated neurotoxicity syndrome; LEMS, Lambert–Eaton myasthenic syndrome; MG, myasthenia gravis; MOGAD, myelin oligodendrocyte glycoprotein antibody-associated disease; MS, multiple sclerosis; N/A, not applicable; NMOSD, neuromyelitis optica spectrum disorder; RA, rheumatoid arthritis; SPS, stiff-person syndrome.

## Basic principles of T cell-based adoptive cell therapy

The most successful and widely used immunotherapy to date is allogeneic hematopoietic stem cell transplantation (allo-HSCT). Following myeloablative chemo- and/or radiotherapy, the patient’s immune system reconstitutes from the donor’s stem cells and ideally eliminates residual malignant cells, while balancing between the so-called “graft-versus-host (GvH) and graft-versus-tumor (GvT) effect.”^25^ Notably, allo-HSCT is also used in AIDs, where the goal is less about GvT effects and more about eliminating a pathogenic immune system. However, due to its substantial treatment-related mortality, largely driven by graft-versus-host disease (GvHD), it is certainly not considered a standard therapy. Owing to that, conventional immunotherapy in AIDs mainly targets nonspecifically the effector B-cell compartment in a time-limited manner via monoclonal antibodies, mechanistically blocking the propagation of the immune dysregulation but not necessarily achieving an “immunological reset,” as will later be discussed. This approach echoes the difference of treatment goals in each patient group, with respect to terminality of disease and preservation of living standards, and is one of the major discussion points underpinning CAR T-cell therapy in nonmalignant diseases.

It is now widely accepted that the transplanted and reconstituted donor T cells are the key mediators of the GvT effect.^26^ A logical refinement, therefore, would be to artificially redirect the action of the infused T-cells against highly specific and well-characterized disease-associated antigens, enabling targeted activity while minimizing side effects such as GvHD. This approach has been exemplified in studies exploring the application of T-cell receptor (TCR)-engineered T-cells that target tumor-specific or tumor-associated antigens.^27^ These biomedical advances are driven by a deep understanding of T-cell function at the molecular level.

The structural and functional basis of T-cell antigen recognition is facilitated by the membrane-bound TCR complex and its co-receptors.^28^ In brief, the TCR is closely associated with invariant CD3 proteins, particularly CD3ζ, which plays a crucial role in intracellular signal transduction upon antigen binding. This occurs through immunoreceptor tyrosine-based activation motifs that initiate a cascade of protein phosphorylation.^29^ Similar to immunoglobulin (Ig) molecules, the TCR repertoire recognizes antigens through gene rearrangements; however, unlike Ig, it does not undergo somatic hypermutation to refine antigen affinity over time. As another key distinction from the Ig on the surface of B-cells, which can bind free-floating antigens in their native form, T cells rely on their TCR to recognize antigens only when they are processed into peptide fragments and presented by major histocompatibility complex (MHC) molecules on the surface of antigen-presenting cells (APCs) or their target cells (i.e., infected, malignant, or foreign cells). Furthermore, following successful antigen binding, the ensuing T-cell immune response depends on so-called “co-stimulatory molecules.” Both, suboptimal and persistent co-stimulation can lead to functional inertness and exhaustion.^30^ A hallmark example of effective co-stimulation enabling T-cell activation and proliferation is the interaction between CD28 on T-cells and CD80 or CD86 on APCs.^31^ In summary, successful T-cell activation hinges on the formation of the immunologic synapse, which depends on the spatiotemporal dynamics of the involved molecules. The goal is to enhance the efficacy of adoptively transferred T cells, especially in overcoming the barriers imposed by the TME.

**Figure 1.**
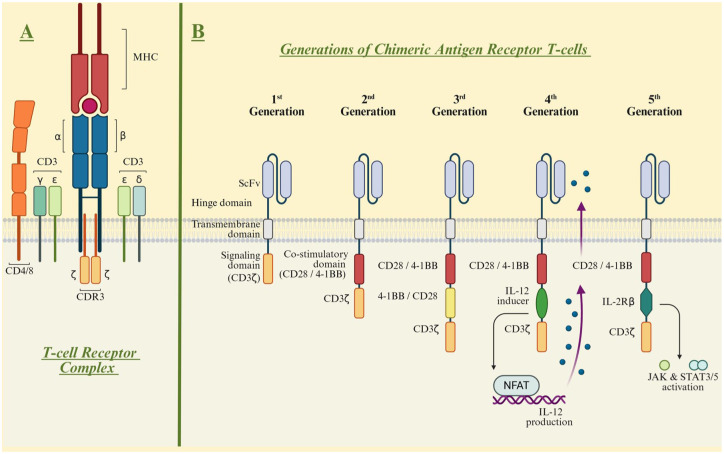
Structure of the TCR and CARs of different generations. (a) The TCR complex consists of α and β chains along with associated CD3-complex chains and co-receptor CD4 or CD8 surface proteins, which interact with an antigen presented by an MHC molecule. This interaction triggers a cascade of downstream signal transduction events, leading to T-cell activation. (b) CAR constructs exemplary for the first to fifth-generation are depicted, highlighting their respective antigen-binding domains, hinge regions, transmembrane domains, and signaling domains (CD3ζ). Co-stimulatory domains may include CD28, 4-1BB, OX-40, ICOS, and CD27. CARs, chimeric antigen receptors; TCR, T cell receptor.

The core principle of the CAR concept is the integration and optimization of these insights (Figure 1(a)).^1^ Unlike conventional TCR-mediated T-cell activation, which relies on three separate components—antigen recognition, activation signaling, and co-stimulation—CARs combine all three functions within a single molecule at fixed ratios. Moreover, antigen recognition occurs in an MHC-independent manner, and the construct is highly modifiable, allowing for extensive optimization. In addition to T cells, other innate immune cells, such as NK-cells or macrophages,^32,33^ can serve as cellular carriers. This highlights the versatility of the approach, though it is not discussed in detail in this review.

## CAR T-cell designs and approved therapies

CARs are engineered type I transmembrane proteins composed of three main domains: an amino-terminal extracellular domain, a transmembrane domain, and a carboxy-terminal intracellular domain. The molecule can be divided into five distinct regions, with the antigen-binding domain being the most critical. This domain is typically a single-chain variable fragment (scFv), which is formed by linking the variable regions of an antibody’s heavy (VH) and light (VL) chains via a flexible peptide linker. Variable regions of heavy-chain-only antibodies (VHHs or nanobodies) represent an interesting alternative offering the advantage of simpler molecular reconfiguration and of accessing an extended array of epitopes.^34^ VHHs are camelid-derived, whereas scFvs can be murine, humanized, or fully human. Among scFvs, fully human variants are preferred as they minimize immunogenicity, enhance persistence, and reduce the risk of antidrug antibody (ADA) responses.^35^ The clinical efficacy of the VHH-based design was proven with the approval of ciltacabtagen-autoleucel (cilta-cel) for r/r multiple myeloma (MM).^36^ Cilta-cel comprises two llama-based VHH regions that bind two different B-cell maturation antigen (BCMA) epitopes. The choice of target antigen for each CAR is based on the disease’s pathophysiology. For currently approved CAR constructs, CD19 was selected due to its uniform expression on B-cell lineage, making it a key target in B-cell lymphomas and acute lymphoblastic leukemia (ALL). Similarly, BCMA was chosen for its specificity to plasma cells in MM.^37^ However, targeting alternative antigens may offer additional therapeutic benefits depending on the clinical context,^38^ or in a potential relapse via antigen escape, as may be the case for GPRC5D-targeting in MM.^39^

The hinge region flexibly connects the antigen-binding domain to the stable transmembrane domain. Collectively, these regions influence CAR T-cell signaling, primarily by determining the receptor’s overall molecular and mechanical properties.^40^ The choice and number of intracellular co-stimulatory domains have been the key determinants in defining CAR T-cell generations. The prototypic first-generation CAR construct engineered by Zelig Eshhar and colleagues included only CD3ζ signaling as its intracellular activation domain (Figure 1(b)).^41^ It enabled in vitro cytotoxicity but failed to sustain T-cell expansion in vivo, limiting its therapeutic efficacy.^42^ Subsequent studies highlighted the critical role of incorporating co-stimulatory domains—such as CD28, 4-1BB, ICOS, and OX40—to achieve full T-cell activation, cytotoxicity, expansion, and persistence.^43^ Clinical and preclinical evidence suggests that the choice of co-stimulatory molecule influences key differences between CAR constructs, including kinetics, metabolic characteristics, toxicity, and efficacy, though no single molecule has been definitively proven superior.^44,45^ CD28-based CARs are associated with faster and more intense signaling, not only driving effector memory differentiation but also increasing the risk of T-cell exhaustion. In contrast, 4-1BB-based CARs promote central memory differentiation through slower, more sustained signaling, enhancing long-term persistence.^46^ Emerging research is also exploring how spatiotemporal dynamics of CAR activation may differ from the physiological TCR-immunological synapse, potentially influencing T-cell subset selection and function.^47–49^ All CAR T-cell therapies approved by the U.S. Food and Drug Administration (FDA) and the European Medicines Agency (EMA) currently belong to the second generation and are approved exclusively for hematologic malignancies.^50–56^ Their characteristics are summarized in Table 2.

**Table 2.** FDA-approved CAR T-cell products (as of February 2025).

Name	axi-cel	brexu-cel	cilta-cel	ide-cel	liso-cel	obe-cel	tisa-cel
Company	Gilead/Kite	Gilead/Kite	J&J	BMS	BMS	Autolus	Novartis
Diseases	DLBCL, HGBCL, PMBCL, FL	MCL, B-ALL	MM	MM	DLBCL, HGBCL, PMBCL, FL, MCL, CLL/SLL	B-ALL	DLBCL, HGBCL, FL, B-ALL
First approval trial	ZUMA-1^3^	ZUMA-2^57^	CARTI-TUDE^58^	KarMMa^59^	TRANS-CEND^60^	FELIX^61^	ELIANA^62^
Target antigen	CD19	CD19	BCMA	BCMA	CD19	CD19	CD19
Antibody-binding domain	FMC63 scFv	FMC63 scFv	dual-VHH	11D5-3 scFv	FMC63 scFv	CAT scFv	FMC63 scFv
Spacer/trans-membrane domain	CD28	CD28	CD8a	CD8a	IgG4/CD28	CD8a	CD8a
Co-stimulatory domain	CD28	CD28	4-1BB	4-1BB	4-1BB	4-1BB	4-1BB
Viral vector	Retrovirus	Retrovirus	Lentivirus	Lentivirus	Lentivirus	Lentivirus	Lentivirus

ALL, acute lymphoblastic leukemia; axi-cel, axicabtagene ciloleucel; AYA, adolescents and young adults; BCMA, B-cell maturation antigen; brexu-cel, brexucabtagene autoleucel; cilta-cel, ciltacabtagene autoleucel; CLL, chronic lymphoblastic leukemia; DLBCL, diffuse large B-cell lymphoma; FL, follicular lymphoma; HGBCL, high-grade B-cell lymphoma; ide-cel, idecabtagene vicleucel; liso-cel, lisocabtagene maraleucel; MCL, mantle cell lymphoma; MM, multiple myeloma; obe-cel, obecabtagene autoleucel; PMBCL, primary mediastinal B-cell lymphoma; scFv, single-chain variable fragment; SLL, small lymphocytic lymphoma; tisacel, tisagenlecleucel; VHH, variable heavy domain of heavy-chain-only antibodies.

The introduction of a second co-stimulatory domain to the now established second-generation CAR backbone led to the development of third-generation CARs. However, this modification did not result in a breakthrough comparable to the transition from first- to second-generation CARs.^63^ The more experimental fourth- and fifth-generation CAR T-cells, though lacking a formal definition, have moved away from previous structural improvements and focus instead on integrating constitutive cytokine secretion and optimizing intracellular signaling at the genetic level.^5,64,65^ Despite their potential, none of these advanced CAR designs have been systematically tested in large-scale clinical studies yet. Particularly in neurological AIDs, most studies employ established second-generation CAR constructs incorporating either CD28 or 4-1BB co-stimulatory domains, while dual-CAR designs are increasingly being explored to improve efficacy.

Therefore, CAR design provides numerous opportunities to modify each domain, allowing for tumor-specific adaptation and precise customization of CAR function. How this modification translates to clinical benefit is a more nuanced subject, though. The roles of CAR construct design, costimulatory domains, and the optimal target antigen remain unresolved and may depend on both disease characteristics and individual patient factors.

Theoretically, and especially for AIDs, fully human CARs should offer advantages in terms of better tolerability and a reduced risk of developing ADAs, which are more common in patients with AIDs.^66^ Up until the point of writing though, all of the above strategies for extracellular domain-construction (i.e., murine,^9,17^ humanized,^16^ and fully human CARs)^8,13^ have been used, so the question which ones are indeed comparatively preferable in AIDs remains open.

Regarding the CAR target molecule, CD19 remains the most equivocal target for B-cell-mediated AIDs. Efficacy data from anti-BCMA-directed CAR T-cell approaches in anti-signal recognition particle (SRP) necrotizing myopathy and MG, though, the latter with a bispecific approach, have also been promising.^16,67^ Other targeted approaches are also being explored, such as in MuSK-positive MG, where chimeric autoantibody receptor T-cells selectively eliminate the B-cell population producing the well-defined pathogenic autoantibody.^68^ However, given that the pathogenic role of B-cells extends beyond autoantibody production, it remains an open question how these approaches will holistically impact B-cell populations and ergo disease pathology. While humoral immunity may not be impaired, a major safety goal, it will be particularly interesting to assess the effectiveness of these treatments. Partly accounting for this, there have been efforts in utilizing fourth-generation CAR T-cells. Three patients with refractory rheumatoid arthritis were recently successfully treated with anti-CD19 CAR T-cells also secreting antibodies against IL-6 and TNFα, which are central to the disease pathophysiology—as well as intricately involved with “cytokine release syndrome” (CRS).^69^ A concurrent engagement of both the pathogenic antibodies and the underlying immune effectors, with a theoretically reduced incidence of CRS complications, is a very appealing structure.

As to the inherent CAR characteristics, one emerging insight is that CAR T-cell persistence does not necessarily appear to be a prerequisite for therapeutic success in AIDs, in contrast to malignant diseases.^70^ Interestingly, even though CAR T-cells in AID patients often persist for only a short period and show signs of diminished functional activity, clinical responses tend to be sustained, suggesting that long-term persistence may not be required for therapeutic efficacy in this setting. This phenomenon may reflect disease-specific dynamics rather than intrinsic T-cell dysfunction. One plausible explanation is that the regenerating hematopoietic compartment, less affected by cytotoxic preconditioning in AIDs than in malignancies, could competitively displace CAR T cells. Additionally, the typically low antigen burden in AIDs may limit antigen-driven expansion and persistence. If confirmed, these factors would suggest that long-term CAR T-cell persistence is not required for therapeutic efficacy in AIDs, thereby shifting construct priorities toward transient but potent immune modulation, such as that achieved with mRNA-based CAR T-cell therapies. Third-generation CAR constructs have indeed shown persistence of up to 2 years in a single AID patient in remission.^71^

**Figure 2.**
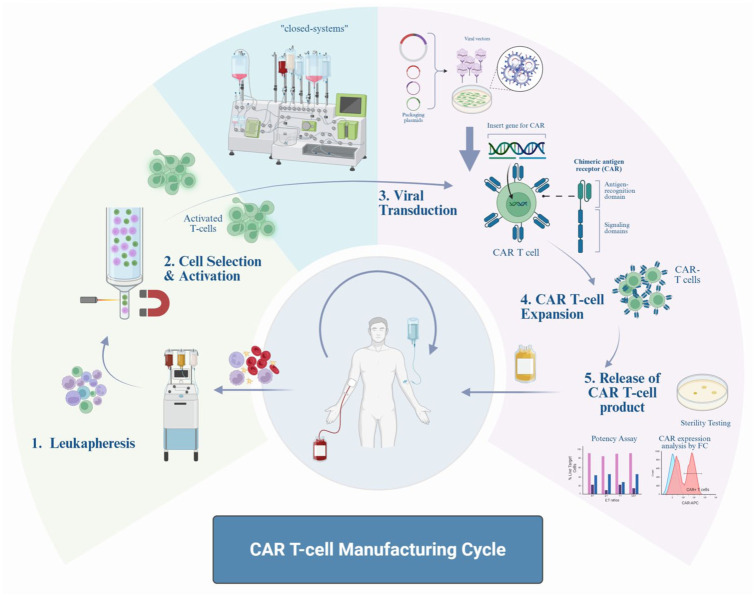
CAR T-cell manufacturing cycle. The process begins with leukapheresis (1), where T-lymphocytes are collected from the patient. These cells are then selected and activated, typically using magnetic beads (2), followed by viral transduction to introduce the CAR construct (3). The modified T cells undergo expansion under specialized culture conditions with cytokines (4). Before reinfusion, the final CAR-T cell product undergoes rigorous quality control to ensure safety and efficacy (5). Together, these steps define the “vein-to-vein” time, representing the entire manufacturing and delivery process. CAR, chimeric antigen receptors.

Further opportunities along the manufacturing process, including allogeneic and RNA-engineered CARs, will be discussed in subsequent paragraphs (Figure 2).

## CAR T-cell-associated toxicities

Since their introduction in clinical trials, CAR T-cell therapies have been linked to a distinct set of complications, many of which have been extensively analyzed in relevant reviews.^72–75^ CAR T-cell therapies are associated with two major toxicities: CRS and immune effector cell-associated neurotoxicity syndrome (ICANS), both of which occur in a significant proportion of treated patients (Table 3).^76^ CRS results from CAR T-cell activation, triggering the release of proinflammatory cytokines such as IL-6, IL-1, and interferon-gamma, which further stimulate myeloid cells to amplify cytokine production, leading to a systemic hyperinflammatory response. The severity of CRS correlates with tumor antigen burden, as a higher number of target antigens drives stronger CAR T-cell activation and more intense cytokine release. While the pathophysiology of CRS is well characterized, ICANS remains less clearly understood. Excessive influx of pro-inflammatory cytokines into the central nervous system (CNS), facilitated by increased blood-brain barrier (BBB) permeability and resulting in microglia activation, has been proposed as key mechanisms, with IL-1 and localized inflammatory axes such CXCL16/CXCR6 and CCR5 overexpression in CD4+ T-cells playing a potential role.^77–80^ In anti-CD19 CAR T-cell therapies, additional on-target, off-tumor effects on CD19-expressing mural cells in the brain may further exacerbate neurotoxicity.^81^ Both CRS and ICANS develop against a backdrop of endothelial inflammation, which may be influenced by disease-specific or genetic predisposition, and prognostic models for risk stratification are currently being validated.^82,83^ Clinical and real-world data suggest that CD28-based co-stimulation is associated with a higher incidence of ICANS in ALL, likely due to faster CAR T-cell expansion postinfusion.^84^ Additional factors implicated in increased CRS and ICANS risk include high tumor burden, which leads to heightened cytokine release, as well as baseline inflammatory status, preexisting neurological conditions, older age, and the intensity of the lymphodepleting regimen.^85–87^ Strategies to mitigate these toxicities are under investigation, including fine-tuning second-generation CAR constructs to optimize intracellular signaling, as well as implementing a fixed CD4^+^ to CD8^+^ T-cell ratio, which may promote a more balanced immune response.^88,89^ Additionally, predictive models are being developed to anticipate adverse events and account for product-specific heterogeneity.^90^ Ongoing research aims to further refine CAR designs and identify biomarkers that can predict toxicity, ultimately enhancing the safety of CAR T-cell therapies without compromising efficacy.

**Table 3.** Incidence of CRS and ICANS in indications of approved CAR T-cell products in clinical trials and real-world conditions.

Name	axi-cel		brexu-cel		cilta-cel		ide-cel		liso-cel		tisa-cel	
Indication	LBCL		MCL		MM		MM		LBCL		LBCL	
	Trial^134^	rw^2^	Trial^57^	rw^135^	Trial^58^	rw^136^	Trial^59^	rw^136^	Trial^60^	rw^137^	Trial^138^	rw^2^
CRS any grade	93%	86.3%	91%	93%	95%	81%	84%	85%	42%	54%	57%	70.6%
CRS grade ⩾ 3	11%	8.2%	15%	10%	4%	10%	5%	>4%	2%	5%	17%	8.9%
ICANS any grade	64%	47.6%	63%	55%	17%	19%	18%	19%	30%	27%	20%	19.9%
ICANS grade ⩾ 3	30%	19.0%	31%	19%	2%	7%	3%	2%	10%	11%	11%	5.8%

No direct comparisons can be inferred among products and between trials and real-world data from the table, owing to diverging patient populations and study designs. The type of previous lines of therapy may differ prior to each product use.

axi-cel, axicabtagene ciloleucel; brexu-cel, brexucabtagene autoleucel; CAR, chimeric antigen receptor; cilta-cel, ciltacabtagene autoleucel; CRS, cytokine release syndromes; ICANS, immune effector cell-associated neurotoxicity syndrome; ide-cel, idecabtagene vicleucel; LBCL, large B-cell lymphoma; liso-cel, lisocabtagene maraleucel; MCL, mantle cell lymphoma; MM, multiple myeloma; rw, real-world data; tisacel, tisagenlecleucel.

Clinically, the presentation of CRS and ICANS can vary significantly, ranging from mild, flu-like symptoms and neuropsychological disturbances to severe cases requiring intensive care unit (ICU) admission.^91^ Notably, laboratory markers and cytokine levels do not directly determine CRS grading, although radiological findings—such as leptomeningeal enhancement, brainstem involvement, or cerebral edema affecting the thalami—may suggest ICANS development in the appropriate clinical context.^92,93^ The typical onset of CRS occurs within the first 1–14 days postinfusion, peaking within the first week depending on the CAR T-cell product used. In contrast, ICANS tends to present slightly later, typically between days 3 and 6, peaking on days 7–8, and resolving within 2–3 weeks. Treatment strategies primarily involve blocking implicated cytokine receptors or signaling cascades, particularly IL-6 and IL-1, with agents such as tocilizumab and anakinra.^94–96^ However, tocilizumab has demonstrated efficacy in managing ICANS primarily when accompanied by CRS. Due to its presumed limited penetration into the cerebrospinal fluid, which may paradoxically result in elevated IL-6 levels within the CNS, some experts recommend avoiding its use in cases of isolated ICANS or when CRS is limited to grade 1.^73,97^ Further clarification of these clinical nuances, as well as the refinement of current treatment strategies, would require evidence from prospective randomized controlled trials.^98^

While ICANS is generally considered to be reversible,^99^ it is still not well known whether there are any long-term effects of CAR T-cell therapy on patient cognition. In a study by Ruark et al.,^100^ 37.5% of patients reported cognitive difficulties in a patient-reported outcome measure-format 1 year after administration of CAR T-cells; this study though, lacked a relevant pre-administration baseline value. Moreover, perceived impaired cognition and objectively measurable cognitive impairment are only weakly correlated,^101^ and yet there is a paucity on studies combining the two. Some of the available studies indicate no significant burden in cognitive outcomes, but owing to small sample sizes and heterogeneous results, more evidence is required for formal conclusions.^99,102,103^

Regarding corticosteroid use, initial concerns about impairing CAR T-cell expansion have been addressed by recent studies, which indicate that early corticosteroid administration can mitigate the initial inflammatory cascade without significantly compromising long-term disease control or survival outcomes.^104^ Nevertheless, any acute intervention for CAR-T-related toxicities may influence the infused CAR T-cell dynamics, potentially affecting the patient’s long-term therapeutic benefit. CRS and ICANS must also be distinguished from immune effector cell-associated hemophagocytic syndrome (IEC-HS), a rare but distinct manifestation of secondary macrophage activation syndrome or hemophagocytic lymphohistiocytosis, that has been primarily observed with T-cell engager (TCE) therapy. IEC-HS typically occurs later in the clinical course and is, by definition, associated with cytopenias, coagulopathy, and/or transaminitis.^105^ In contrast, the much more common phenomenon of isolated cytopenias, collectively termed immune effector cell-associated hematotoxicity (IACHT), is considered to be multifactorial, resulting from both the lymphodepleting conditioning regimen and the immune effector cell therapy itself. The CAR-HEMATOTOX scoring system, which incorporates baseline cytopenia and inflammatory markers, has been validated across all currently approved CAR T-cell indications and serves as a practical tool for identifying patients at risk for prolonged (i.e., >90 days) hematotoxicity.^106,107^ Management of IACHT includes supportive care, such as growth factor administration, evaluation of infection prophylaxis duration, and determination of optimal vaccination timepoints.^108–111^ However, severe or persistent cases may necessitate an autologous stem cell boost if available and even rescue HSCT.^112^ A recently published study comparing hematotoxicity in some of the first patients with systemic lupus erythematosus (SLE) treated with CAR T-cell therapy to established data from B-cell lymphoma patients shows that the neutrophil lineage is primarily affected in both groups; however, the duration of hematotoxicity in SLE patients appears to be several months shorter than in those with malignancies.^113^

The on-target, off-tumor effect refers to the unintended lysis of normal cells that physiologically express the targeted surface antigen.^114^ In CD19- and BCMA-targeted therapies, B cells are partially and often long-term depleted, leading to impairment of humoral immunity.^115^ In cases of severe hypogammaglobulinemia and/or recurrent infections, immunoglobulin substitution is administered.^110^ However, it is important to note that infections represent the most significant contributor to nonrelapse-related mortality (NRM) in CAR T-cell therapy,^116^ whereby the role of CMV-reactivation is also currently under investigation.^117^

The toxicity profile of CAR T-cell therapy will inevitably vary depending on the target antigen and the affected cell type. For instance, particularly for BCMA-directed CAR T-cell therapies, and depending on the product, delayed neurotoxicity has been reported, reaching cumulatively up to 10%, and mainly include cranial nerve palsies, parkinsonism, and polyneuropathies.^118–120^ Additionally, the underlying disease itself will influence the nature and severity of treatment-related side effects. So far, most clinical experience has been gathered from B-cell-depleting CAR T-cell therapies in B-cell malignancies, and whether these findings can be directly extrapolated to other diseases remains uncertain.^75^

Another potential risk that warrants further investigation is the development of secondary, particularly myeloid, malignancies. The NRM bestowed by secondary malignancies may range from 5.8% to 7.8% in recent meta-analyses,^116,121^ up to 33% in a different large cohort,^122^ depending on observation time and on how the primary competing NRM risk, namely infections, are accounted for. Reports from FDA support the prospective emergence of secondary neoplasms at a nonnegligible rate post-CAR T-cell treatment.^123^ These findings however, require careful extrapolation to AIDs, since their mechanism may not only be accounted for by the singular chemotherapy conditioning and the product infusion alone. One possible underlying mechanism is clonal hematopoiesis induced by prior treatment modalities and lymphodepleting regimens,^124^ which would not necessarily be indicative of the population at hand. Even though there is growing evidence that clonal hematopoiesis of indeterminate significance may indeed be more prevalent in AID-patients compared to the general population, this need not necessarily be true compared to cancer patients.^125,126^ The increased preexisting genomic hits and germline mutations in this patient group may further confound the extrapolation.^127^ Conversely, the integration of genetic sequences into the host genome—even when restricted to T cells and despite advances in vector safety—carries the potential risk of insertional mutagenesis, which may drive malignant transformation. Compared with eventual secondary myeloid malignancies, their incidence appears to be much smaller, but a more profound causality may be inferred.^128,129^ Recent reports have documented such cases in a small number of patients receiving both anti-CD19 and anti-BCMA CAR T-cell therapies. Nevertheless, there is an a priori increased incidence of secondary T-cell malignancies in B-cell neoplasm patients, and vice versa, somewhat obscuring the aforementioned causality.^130^ Cumulatively, this highlights the need for a deeper understanding of the contributing factors and the development of improved monitoring and early detection strategies.^131^ More recently, an apparent AID-specific side-effect of CAR T-therapy has been described and termed “Local immune effector cell-associated toxicity syndrome” (LICATS).^132^ In one pool of 39 patients receiving CAR T-cell therapy, 30 of them showed evidence of an organ-specific inflammatory reaction exclusively in one or more of the initially involved disease sites (e.g., kidney, skin, joints, muscles), that was irrespective of any underlying CRS-diagnosis. All of the events occurred during the B-cell aplasia period (80% of them within the first month postinfusion) and resolved either in a self-limiting manner or with a short treatment course of corticosteroids. More specifically, 20 patients necessitated no corticosteroid usage (being termed as LICATS Grade 1), and all others necessitated less than 67 mg of prednisolone dose (maximum cumulative dose of 244 mg) for complete symptom resolution. The occurrence of the events in a per se still immunocompromised organism, as well as their way of resolution speak against an AID-flare; nevertheless, histological correlates are reported in only four patients, as the work-up consisted mainly on clinical evidence, and laboratory and/or imaging studies. The formal description of LICATS in organ-specific AIDs draws parallels with the nonspecific inflammatory changes observed in the lymph nodes of some lymphoma patients, which indeed do not signify disease progression. Moreover, similar findings were also observed in two different refractory SEL patient cohorts, where 4 out of 19 patients had documented reactions consistent with LICATS, strengthening its introduction. Of note though, is the description of two events (diarrhea and arthritis) occurring in nonpreviously affected regions.^133^ Attempting to ascertain the exact scope of LICATS, as well as studying any overarching pathophysiology of nonCRS-related postinfusion immune reactions in AIDs remains an active focus of research.

As CAR T-cell therapy continues to evolve, with expanding indications and the development of next-generation products, it is highly likely that risk assessment strategies will need to be adapted to account for new toxicities and patient-specific factors.

## Manufacturing, optimization, and clinical implementation of CAR T-cell therapy: Lessons from hematological malignancies

The process of CAR T-cell therapy, whether in a commercial or academic setting, involves several critical and highly coordinated steps, all of which must adhere to good manufacturing practices.^139–141^ It begins with the collection and transportation of donor material, followed by a rapid and precise manufacturing process, transportation to the treating facility, administration to the patient, and continuous postinfusion care with the FDA suggesting a life-long follow-up (Figure 2). Since CAR T-cell therapies are administered on an individual-patient basis, their production differs fundamentally from conventional bulk pharmaceutical manufacturing. A key aspect of this process is traceability, ensuring that each product follows a stand-alone chain of identity for the patient and a chain of custody for the product. The process involves a multidisciplinary team of laboratory, logistics, and clinical personnel, highlighting opportunities for optimization across these fields.^142^ Lessons from CAR T-cell therapy in hematologic malignancies have provided valuable insights.

Clinical experience suggests that patients with lower baseline tumor burden achieve deeper and more durable responses. This underscores the importance of early patient referral to accredited institutions and the expansion of clinical trials to explore broader indications and earlier lines of therapy. Whether this also holds true for AIDs is under investigation.

Although patient selection is primarily guided by the eligibility criteria of pivotal trials leading to product approval, CAR T-cell therapy remains highly individualized, with no absolute fitness exclusion criteria.^143^ Notably, patients with AIDs coexisting with malignancies were typically excluded in clinical trials—due to concerns about disease flares, impaired T-cell function under chronic immunosuppression, and the risk of confounding adverse events. Moreover, concurrent immunosuppressive therapy may interfere with CAR T-cell expansion and persistence, complicating the assessment of safety and efficacy endpoints. However, early real-world data from retrospective analyses suggest that CAR T-cell therapy is well tolerated in patients with concurrent AIDs, showing comparable toxicity profiles and antitumor efficacy to those without such comorbidities. Notably, some reports have indicated biochemical or clinical improvements in AID activity post-CAR T-cell infusion.^144^

Instead, cell quality—including the number, ratio, and transduction efficiency of isolated T-cells—is prioritized to proceed with the therapy, as these factors directly impact therapeutic efficacy. Additionally, both the type of prior therapy and the underlying disease can influence these parameters.^145,146^ In patients with malignancies, adherence to predefined treatment washout, both prior to leukapheresis and CAR T-cell infusion, is typically feasible. In contrast, patients with AIDs are often maintained on continuous immunosuppression, which presents unique challenges for CAR T-cell manufacturing and therapeutic efficacy. Immunosuppressive agents such as mycophenolate mofetil, cyclosporine, or high-dose corticosteroids can impair T-cell function and expansion, potentially compromising both the CAR T-cell product and its in vivo performance.

In clinical practice, tapering or temporarily discontinuing T-cell-modulating agents, when disease control allows, is generally recommended. For corticosteroids, a reduction to ⩽5–10 mg/day of prednisolone equivalent is often feasible and may serve as a bridging strategy. Alternatively, treatment-free intervals of at least 3–7 days, depending on the elimination half-life of the respective agents, are encouraged prior to leukapheresis or infusion where clinically feasible. However, such modifications carry a risk of disease flares, and tapering should therefore be performed cautiously and individually adapted.

Similar considerations apply to the postinfusion phase: immunosuppressive agents administered to control flares may also interfere with CAR T-cell persistence and activity. These aspects are particularly relevant not only for autologous CAR T-cell therapies but also for allogeneic and in vivo gene-engineering approaches.

The manufacturing process consists of distinct steps that have been largely streamlined, and in part automated. These include cell sorting (particularly for specific T-cell subpopulations),^147^ polyclonal T-cell activation (usually via antibody-coated microbeads), gene transduction, and subsequent expansion,^148^ all of which have the potential to actively influence the CAR T-cell characteristics in vivo.^139^ Beginning with leukapheresis, it is typically aimed to collect a minimum of 0.6 × 10^9^/L cells, though the required lymphocyte threshold may vary by manufacturer. Recent European Society for Blood and Marrow Transplantation/European Haematology Association guidelines provide further recommendations on this aspect.^111^ Leukapheresis products enriched with less-differentiated T-cells—such as naïve (*T*_n_), stem cell like memory (*T*_SCM_), or CD8^+^CD45RO^neg^CD27^+^ T-cells displaying both effector and memory cell characteristics—have been associated with superior antitumor control and improved clinical outcomes in MM and chronic lymphocytic leukemia.^149–151^ CAR T-cell persistence is a major determinant of long-term therapeutic responses, at least in hematologic malignancies.^152^ To reduce immunotoxicity and improve outcomes, separate processing of purified CD4^+^ and CD8^+^ T-cells with fixed 1:1 CD4^+^ to CD8^+^ CAR T-cell ratio is already implemented for certain approved products.^153,154^ This approach is based on the observation that CD4^+^ CAR T-cells not only serve as key effector cells but are also the primary drivers of toxicities such as the CRS.^154^

A key challenge in CAR T-cell manufacturing is designing and generating the vector for CAR transgene integration. All currently approved CAR T-cell products rely on viral vectors (either lentiviral or retroviral), as neither method has demonstrated a decisive advantage,^155^ and with the caveat of high cost and potential risk of insertional mutagenesis. CRISPR/Cas9 technology offers more precise and multiplexed gene editing, potentially allowing for the fine-tuning of next-generation CAR constructs via targeted knock-in and/or knock-out^156^; this would allow for allogeneic products for AIDs.

Shorter vein-to-vein times have been correlated with improved clinical outcomes.^157,158^ However, major logistical and regulatory hurdles remain, spanning transport, approval processes, manufacturing, and prerelease quality control testing.^159,160^ The introduction of “off-the-shelf” allogeneic CAR T-cell products could revolutionize the field, although previous efforts were limited by issues such as poor persistence and T-cell exhaustion due to concurrent TCR and CAR engagement. Ongoing trials are exploring ways to overcome these limitations,^161,162^ with successful steps also in the AID-domain, as will later be discussed.

One of the major causes of CAR T-cell failure in malignancies is disease progression during the approval and manufacturing period. In many cases, patients receive bridging therapy to control disease before undergoing lymphodepleting chemotherapy prior to CAR T-cell infusion.^163^ While bridging therapy may further immunosuppress patients and impair T-cell regeneration, lymphodepletion serves an opposite role by creating a niche for CAR T-cell engraftment and rapid expansion, a process termed homeostatic proliferation.^164^ The most commonly used lymphodepleting agents are fludarabine and cyclophosphamide, although these regimens are not universally optimal.^165^ One of the most commonly used alternatives is bendamustine, which has a favorable toxicity profile.^166^ All FDA- and EMA-approved CAR T-cell therapies require preconditioning regimens. Extrapolating from the debated corticosteroid thresholds used in the treatment and prophylaxis of CRS and ICANS, a similar rationale applies to AID-patients receiving immunosuppressive therapy: tapering is often required to avoid compromising CAR T-cell expansion and persistence.

Following infusion, patients undergo distinct follow-up phases to monitor acute, mid-, and long-term complications. The first phase spans the initial 28 days, primarily focusing on monitoring for CRS, ICANS, and infections, necessitating access to an ICU. Up to day 100, the main concerns shift to immune recovery and reconstitution, which influence susceptibility to opportunistic infections, viral reactivation, and CAR-T cell persistence. Beyond this period, the emphasis transitions to long-term disease control, prolonged cytopenia, and organ function assessment, with patient visits occurring at progressively longer intervals.^73^ Comprehensive safety monitoring and optimized follow-up protocols are essential for improving real-world patient outcomes.

Even with an optimal CAR T-cell product, treatment failure may occur due to several factors: postinfusion CAR T-cell dysfunction, which may arise from cell-intrinsic or TME-related factors, severe toxicities leading to nondisease-related mortality, a hostile TME preventing infiltration (especially in solid tumors),^167^ and tumor-intrinsic factors such as antigen loss and multilineage heterogeneity. Implemented strategies would aim to enhance CAR T-cell persistence, counteract immunosuppressive signals, and improve tumor infiltration, ultimately increasing therapeutic efficacy. Combination strategies involving CAR T-cells with other immunotherapies—such as checkpoint inhibitors or bispecific antibodies—are also under evaluation.^168–170^ These strategies are particularly relevant as CAR T-cell therapies are being moved earlier within the treatment algorithms. Ongoing trials and post hoc analyses will be essential in shaping optimal decision-making strategies for integrating CAR T-cell therapy into clinical practice.

Summarizing, from a neurologist’s perspective, key considerations center on the individualized nature of CAR T-cell manufacturing. The patient’s “journey” typically begins with inclusion in a clinical trial or treatment under exempt approval. Prior therapies may affect T-cell quality at collection, while concurrent immunosuppression can influence in vivo CAR T-cell expansion and persistence. Manufacturing processes are largely standardized, drawing from hematologic experience (e.g., use of less-differentiated T cells, fixed CD4:CD8 ratios). Efforts toward allogeneic CAR platforms aim to reduce vein-to-vein time and ensure consistent product quality—an approach increasingly relevant for autoimmune indications. Close and long-term follow-up remains essential to manage both known and emerging complications.

## Early clinical experience with B-cell-depleting CAR T-cell therapy in AIDs

**Figure 3.**
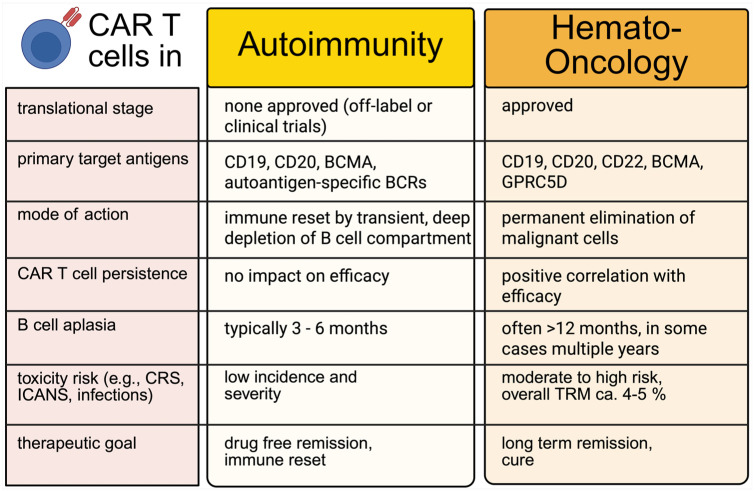
Key differences in the clinical application of CAR T cell therapy in autoimmunity versus hemato-oncology across translational, mechanistic, and therapeutic domains. While CAR T cells in hematologic malignancies aim for long-term persistence and cytotoxic elimination of malignant cells, their use in autoimmunity is characterized by transient B cell depletion to achieve immune reset and drug-free remission. These divergent goals shape distinct profiles regarding persistence, toxicity, and regulatory development. Notably, early clinical experience in autoimmunity suggests a more favorable tolerability profile to date, with lower incidence and severity of adverse events such as CRS or ICANS. BCR, B cell receptor; CAR, chimeric antigen receptors; CRS, cytokine release syndrome; ICANS, immune effector cell-associated neurotoxicity syndrome; TRM, treatment-related mortality.

Early experience with the repurposing of CAR T-cell therapy beyond oncology has already revolutionized the therapeutic outlook in AIDs and generated significant scientific discourse. The unique characteristics of CAR T cells, particularly their ability to achieve deep and sustained depletion of target cells, make them a promising candidate for the treatment of B-cell-mediated AIDs (Figure 3). Conventional therapeutic approaches for controlling pathological B-cell responses include the use of monoclonal antibodies, such as the anti-CD20 antibody rituximab, to eliminate B-cells through antibody-dependent cytotoxicity, antibody-dependent phagocytosis, and complement-mediated cytotoxicity.^171,172^ Additional strategies involve blocking B-cell activation with agents such as anti-BAFF antibodies, direct antibody removal via apheresis, or the administration of nonspecific immunosuppressants, including corticosteroids.^173–175^ More recently, noncell based attempts to engage the T-cell compartment have included the use of TCEs in MG. However, these approaches require long-term administration and may not be sufficient to target tissue-resident B-cells.^176,177^ CAR T-cell therapy has the potential to overcome these limitations by engaging multiple immune compartments, infiltrating tissues, and acting autonomously. Indeed, CD-20 mAbs and CD-19/CD-3 TCEs in patients with AIDs, despite their promising major clinical utility,^178^ have achieved full depletion of B-cells or disruption of the follicular architecture in excised lymph nodes—effects that have been seen with CAR T-cell therapy.^179^

The first report came from a case study of a young patient with SLE who was successfully treated with anti-CD19 CAR T-cells and remained therapy- and disease-free 3 years posttreatment.^180^ This was followed by a case series in five SLE patients, demonstrating a rapid (within 3 months) and sustained remission.^181^ Notably, B-cell regeneration occurred within 3–4 months without disease recurrence, while the circulating CAR T-cell population contracted relatively quickly. This case series was later expanded to include patients with systemic sclerosis (SSc) and idiopathic inflammatory myopathies (IIM), where the observed positive effects were maintained even with longer follow-up periods.^70^ A growing body of research now demonstrates that CAR T-cell-mediated B-cell depletion is a promising therapeutic approach for AIDs within the rheumatologic spectrum. Consequently, a large number of studies have been initiated, particularly considering that the incidence of AIDs—and thus the number of potential patients—is significantly higher than that of malignant diseases.^182^ The observation that B-cell reconstitution did not lead to disease relapse led to the concept of an “immunological reset.” The exact mechanisms by which CAR T-cells mediate this reset in AIDs remains to be elucidated, but early data provide important insights.^183,184^ B-cell reconstitution occurs significantly earlier in AID patients treated with CAR T-cells compared to those with hematologic malignancies. Interestingly, the disease does not relapse alongside B-cell reconstitution, in contrast to what is often observed after treatment with monoclonal anti-CD20 antibodies.^185^ This early regeneration may be related to the faster contraction of CAR T-cells, which, in turn, is likely due to the rapid elimination of the target antigen. Additionally, the reconstituting immune system following lymphodepletion appears to be more competitive in AID patients than in heavily pretreated individuals with malignant diseases. The regenerated B-cells are predominantly naïve, while pathologic autoantibodies are often, but not always, completely depleted. Importantly, the extent of antibody depletion varies by underlying disease—for instance, in SLE, autoantibody levels are typically fully depleted, whereas in other conditions, residual pathogenic antibodies may persist.^11,181^ Nonetheless, clinical remission can still be achieved even in cases where autoantibodies are not entirely eliminated, further supporting the notion that the pathological role of B-cells extends beyond autoantibody production. In addition to generating autoantibodies, B-cells contribute to pro-inflammatory cytokine production, immune homeostasis, and antigen presentation, all of which play a role in AID pathogenesis.^171^ Interestingly, the impact on humoral immunity is less profound than initially expected. This can be attributed, in part, to the relatively short duration of B-cell aplasia and, in the case of anti-CD19-directed CAR T-cell therapies, to the fact that CD19-negative long-lived plasma cells are not eradicated. As a result, protective vaccine titers are largely preserved in most patients. These observations also provide valuable insights into the pathogenic role of different B-cell populations, particularly regarding the contribution of long-lived plasma cells to the maintenance of AIDs.^115,186^ Interestingly, anti-BCMA CAR T-cell therapies, which target BCMA-expressing plasma cells, including long-lived plasma cells, have also shown encouraging clinical outcomes, further supporting the role of (possibly short-lived) plasma cell depletion in AID treatment.

Regarding their use in neuroimmunological B-cell-mediated AIDs, experience from CNS lymphomas has clearly demonstrated that CAR T-cells can cross the BBB. Importantly, CNS involvement does not appear to be associated with a significantly increased toxicity profile, suggesting that CAR T-cell therapy may be a viable approach for neurological AIDs without an unacceptable risk of neurotoxicity.^187^ Initial experiences with CAR T-cell therapy in neuroimmune disorders have now been reported, including in peripheral nervous system diseases such as MG and Lambert–Eaton syndrome, as well as CNS diseases like MS and stiff person syndrome.^10,13,15^ In treatment-refractory cases, B-cell- and plasma cell-depleting CAR T-cell therapies have already been administered, with promising early results.^144,188^ In a relapsing case of concurrent MS and autoimmune arthritis following CAR T-cell therapy, the patient safely underwent rescue autologous stem cell transplantation, which led to a transient decline in the T-cell compartment but no measurable impact on B-cells.^22^ However, these findings require further validation in the numerous ongoing clinical trials.^189^

The use of CAR T-cells in AIDs presents significant safety considerations, particularly because morbidity and mortality in AIDs differ from those in neoplastic diseases. Preliminary data regarding acute toxicities such as CRS and ICANS are highly promising, likely due to the lower antigen burden compared to neoplasms. However, longer follow-up is required, particularly with respect to secondary neoplasms. Two emerging strategies—mRNA-based and allogeneic (allo-)CAR T-cell therapies—have shown promise in enhancing safety for patients with AIDs, particularly in early-stage studies.

Rather than using DNA vectors for stable CAR expression, Granit et al.^9^ engineered CD8+ T cells via mRNA, enabling controlled ex vivo proliferation. This approach allows for predictable cell kinetics, temporally limited effector function, and transient CAR expression—advantages that reduce the risk of acute toxicities and eliminate concerns related to genomic integration. Notably, it also circumvents the need for lymphodepletion, thereby sparing patients from the toxic effects of chemotherapy. However, the transient nature of expression raises questions about long-term efficacy, and repeated dosing would likely drive-up manufacturing costs, underscoring the need for optimized production platforms.

Interestingly enough, a recent preclinical study aims to transition the CAR T-cell engineering from ex vivo to in vivo.^190^ Building on this concept, CD8-targeted lipid nanoparticles have been developed to deliver CD19-CAR mRNA in vivo, offering the potential for similarly specific cytotoxic activity through repeated, temporally controlled dosing.

Moreover, a key concern regarding the use of autologous CAR T-cell products in AIDs is the genetic modification of potentially autoreactive T-cells. One potential strategy to mitigate this risk and improve accessibility is the use of allo-CAR T-cells. These “off-the-shelf” products could be generated from a single healthy donor to treat multiple patients, offering not only cost advantages but also improvements in scalability, logistics, and manufacturing efficiency. Collectively, these features could substantially increase global accessibility to CAR T-cell therapies. To mitigate the risk of GvHD and enhance persistence, allogeneic T-cells can be genetically modified using CRISPR/Cas9-based editing, for example, by knocking out the TCR to eliminate GvHD potential, and MHC molecules to reduce host-mediated rejection. Data from case series using anti-CD19 allo-CAR T cells in SSc and IIM with this approach have been promising in terms of both efficacy and tolerability.^191^

In an effort to leverage the low immunogenicity of NK-cells, an alternative strategy employed human-induced pluripotent stem cells to generate CAR NK-cells with enhanced safety and specificity.^192^ To prevent recognition by host T cells, MHC molecules were knocked out. Cytotoxic activity was restricted exclusively to the CAR-mediated pathway, and activation was further enhanced via IL-2 receptor signaling. A safety switch targeting EGFR was also integrated, allowing depletion of the CAR NK cells if needed. The resulting bispecific anti-BCMA/CD19 CAR NK cells induced a favorable therapeutic response lasting over 6 months in a patient with poly-refractory SSc.^192^

Taken together, leveraging deep and sustained B-cell depletion appears to be a promising and viable therapeutic strategy for AIDs—particularly in severe neurological manifestations—offering a potential alternative to conventional immunosuppressive approaches. Robust clinical studies will be essential to define the safety and efficacy profiles of these emerging therapies, while translational efforts will be key to addressing both anticipated and unforeseen challenges. This remains a rapidly evolving field, with ongoing research expected to shape future clinical practice.

This study is a narrative review of the literature, which inherently limits its scope compared to a systematic review. Due to the currently limited availability of individual patient data, which are mainly derived from case reports and series, and the fact that most major clinical trials are still ongoing, with only sparse interim results published, a precise estimation of adverse event type and incidence, such as that provided by a meta-analysis, is not yet feasible. Likewise, meaningful extrapolation of treatment effects is constrained, given the heterogeneous nature, pathophysiology, and natural history of the neurological AIDs at hand—much less if exact parallels were to be drawn with rheumatological or hematological AIDs.

## Conclusion

Adoptive cellular therapies, particularly CAR-based approaches, are increasingly recognized as powerful tools for modulating the immune system across a broad spectrum of diseases beyond oncology. In the context of treatment-refractory AIDs, preliminary data suggest both safety and efficacy. However, the complexity of immunological disorders, their incomplete pathophysiological understanding, and their heterogeneous manifestations present challenges for clinical research. Ongoing studies may be constrained by these nuances, as well as by initially small patient populations. Well-designed future clinical trials will be essential to validate the curative potential of CAR T-cell therapy in AIDs, refine patient selection criteria, and assess long-term safety and durability of responses.

The extensive experience gained from treating hematologic malignancies—where tens of thousands of patients have received CAR T-cell therapy—provides valuable insights that can help guide the scientific community in this emerging field. While current studies in AIDs are still in their early stages, they hold the potential to transform the therapeutic landscape. By leveraging lessons from hemato-oncology, we can continue advancing our understanding and application of CAR T-cell therapy in AIDs, fostering cautious but well-founded optimism about its future clinical impact.

## Declarations

**Ethics approval and consent to participate:** Not applicable.

**Consent for publication:** Not applicable.

**Availability of data and materials:** Not applicable.
